# A high-conductance chemo-optogenetic system based on the vertebrate channel Trpa1b

**DOI:** 10.1038/s41598-017-11791-z

**Published:** 2017-09-19

**Authors:** Pui-Ying Lam, Suresh K. Mendu, Robert W. Mills, Baohui Zheng, Hugo Padilla, David J. Milan, Bimal N. Desai, Randall T. Peterson

**Affiliations:** 1 Cardiovascular Research Center, Department of Medicine, Massachusetts General Hospital, Harvard Medical School, Charlestown, MA 02129 USA; 2 Department of Systems Biology, Harvard Medical School, Boston, MA 02115 USA; 3 Broad Institute, Cambridge, MA 02142 USA; 4 Department of Pharmacology and Toxicology, College of Pharmacy, University of Utah, Salt Lake City, UT 84112 USA; 5 Department of Pharmacology, University of Virginia School of Medicine, Charlottesville, VA 22908 USA

## Abstract

Optogenetics is a powerful research approach that allows localized optical modulation of selected cells within an animal via the expression of genetically encoded photo-excitable ion channels. Commonly used optogenetic techniques rely on the expression of microbial opsin variants, which have many excellent features but suffer from various degrees of blue spectral overlap and limited channel conductance. Here, we expand the optogenetics toolbox in the form of a tunable, high-conductance vertebrate cation channel, zTrpa1b, coupled with photo-activated channel ligands, such as optovin and 4g6. Our results demonstrate that zTrpa1b/ligand pairing offers high light sensitivity, millisecond-scale response latency *in vivo*, as well as adjustable channel off latency. Exogenous *in vivo* expression of zTrpa1b in sensory neurons allowed subcellular photo-activation, enabling light-dependent motor control. zTrpa1b/ligand was also suitable for cardiomyocyte pacing, as shown in experiments performed on zebrafish hearts *in vivo* as well as in human stem cell-derived cardiomyocytes *in vitro*. Therefore, zTrpa1b/optovin represents a novel tool for flexible, high-conductance optogenetics.

## Introduction

Over the last decade, optogenetics as a versatile optical interrogation tool has proven to be a transformative approach to various fields of basic research. Light-mediated control of many biological processes has been demonstrated, and optical control of channel function has been of particularly intense interest. Since the first discovery of channelrhodopsin, the optogenetic toolbox for controlling channel function has grown and diversified^1–4^. New tools have included channelrhodopsin variants with altered optical and physiological properties^5–13^ as well as photo-switchable ligands for vertebrate channels^14–21^. The commonly used optogenetic techniques involve the expression of microbial opsins, which have many excellent features but all suffer from various degrees of blue spectral overlap and limited channel conductance. The blue spectral overlap has made it challenging to combine more than one optogenetic receptor in a single experiment, because it is difficult to find illumination conditions for two receptors that do not cross-activate each other. Meanwhile, the limited channel conductance of the opsins (e.g. <1 pS for ChR2)^22,23^ requires that many channels be open simultaneously to trigger cellular depolarization, and therefore most opsins must be expressed at high concentrations within a cell, which can alter cell physiology and viability. Due to these reasons, optogenetic actuators with red-shifted action spectra^7,10,24–26^, or operating at lower light power have been created^27^. Nevertheless, further expansion of the optogenetic toolkit, with systems that offer different spectral properties and increased conductance, will continue to improve the utility and flexibility of optogenetics.

Chemo-optogenetic channels (optopharmacology or optogenetic pharmacology tools) have been under rapid development (see review ref.^28^). High conductance channels, such as nicotinic acetylcholine receptors LinAChRs or light-gated glutamate receptors LiGluRs, have been engineered to be photo-controllable^29–32^. Another attractive candidate for a high-conductance chemo-optogenetic channel is TRPA1. TRPA1 is a member of the transient receptor potential (TRP) channel family^33,34^ and exhibits a roughly 1000X higher conductance than channelrhodopsin^35^. TRPA1 is activated by a wide range of natural products, including allyl isothiocyanate (AITC), cinnamaldehyde and allicin. Most of the known TRPA1 ligands activate TRPA1 via covalent modification of cysteines in the N-terminus^36^ but are not photo-sensitive. However, the previously identified photo-activated ligand optovin has been shown to activate endogenous Trpa1b upon violet light stimulation in zebrafish^37^. The Trauner group has previously succeeded in converting a different TRP channel, TRPV1, into a photoreceptor with a synthetic photoswitch that has the ability to control neuronal activity^38^. We therefore reasoned that TRPA1 and optovin could form the backbone of a high-conductance chemo-optogenetic system and set out to develop and test the capabilities of such a system.

Here we characterize several TRPA1 homologs and numerous optovin analogs in a variety of neuronal and non-neuronal contexts. Chemical modification of optovin led to several derivatives with differing channel off rates. The best channel/ligand combinations exhibit high light sensitivity as well as millisecond scale activation latency *in vivo*. In addition, we show that exogenous expression of zTrpa1b in neurons and cardiomyocytes enables photo-dependent cell depolarization that activates physiological cell functions *in vitro* and *in vivo*.

These data suggest that zTrpa1b/ligand pairing, as a robust chemo-optogenetic actuator, can be used in both neuronal and non-neuronal excitable cells. Features such as its violet light activation, high unitary conductance, high light sensitivity, Ca^2+^ selectivity, and adjustable channel off rate make it a complementary alternative to the existing chemo-optogenetic tools.

## Results

### Activation of endogenous Trpa1b with optovin and light

**Figure 1:**
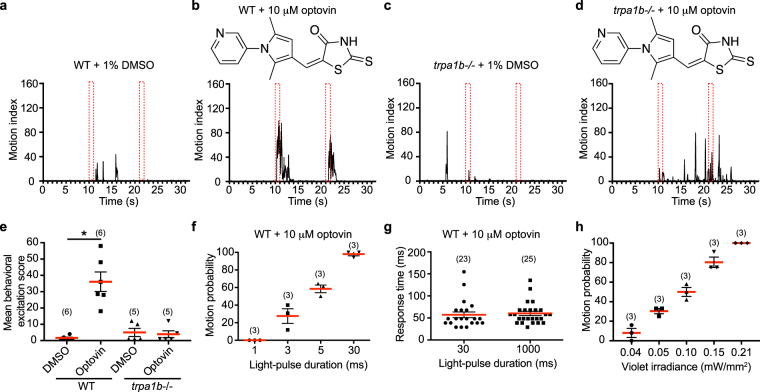
Endogenous zebrafish Trpa1b channel response to optovin. (**a**,**b**) Photomotor response (PMR) assay on WT larvae at 3 dpf. Larvae were pre-treated with 1% DMSO control (**a**) or 10 μM optovin (**b**) for 1 h before the assay. Representative PMR graphs, from >3 sets of experiments, plotting the motion of larvae against time. The red dotted rectangles indicate the timing of 1 s light pulses. (**c**,**d**) Representative PMR graphs, from >3 sets of experiments, plotting the motion of the larvae against time in *trpa1b* mutant (*trpa1b*−/−) larvae pretreated with DMSO control (**c**) or 10 μM optovin (**d**). (**e**) Bar graph showing the average behavioral response of the different treatment groups shown in (**a**–**d**). Values are means ± SEM from at least 3 experiments. Only WT larvae with optovin treatment showed motor response to light. *p < 0.005. (**f**) PMR assays with various stimulus duration were tested on WT larvae at 3 dpf pretreated with 10 μM optovin. Violet light at 1.56 mW/mm^2^ at the indicated light-pulse duration was used. Values are means ± SEM from 3 experiments. (**g**) Larvae were photo-activated for either 30 ms (n = 21) or 1000 ms (n = 25) and the first motion response time from the beginning of light pulse was calculated. Each data point indicates the response time of an individual larva. Data are representative from 3 experiments. (**h**) PMR assay with various intensity of light stimulation as indicated. Light pulse of 30 ms at 415 nm was used. Data were collected from 3 sets of experiments.

We have previously identified optovin as a reversible photo-activated ligand of endogenous zebrafish TRPA1^37^. Refining the previously used photomotor response (PMR) assay, WT zebrafish larvae at a later developmental stage of 3 days post fertilization (dpf) were used. This can maximize small molecule penetration without the hindrance of a chorion, as well as minimize the unique intrinsic light response of earlier stage larvae to the first light exposure. Larvae were subjected to two 1-second flashes of white light with 10 second dark periods between the two flashes. The motion of the larvae in response to light was recorded and quantified. At this stage of development, WT larvae are non-responsive to light (Fig. 1a,e). Optovin rendered zebrafish larvae responsive to light in the PMR assay (Fig. 1b,e). The zTrpa1b cation channel was identified as the endogenous target, as *trpa1b* mutants failed to respond to optovin/light activation (Fig. 1c,d,e). We then tested if violet light pulses shorter than 1 second were sufficient to activate this optovin/zTrpa1b driven motor response by performing photoactivation with different light-pulse durations. Larvae reacted to light activation with light-pulses as short as 3 ms (Fig. 1f). The probability for larvae to respond to light increased as the duration of light stimulation increased, with a 100% response rate using a 30 ms light-pulse duration (Fig. 1f). When larvae were activated with a 30 ms or 1000 ms light pulse, there was no significant difference between their corresponding initial response time (Fig. 1g). This suggested that 30 ms light activation was sufficient to trigger a maximum motion response. The biological response triggered by zTrpa1b light activation was very rapid, with a delay in motion in millisecond scale from the introduction of light. The minimum light required for channel activation was determined by adjusting the light intensity used for photoactivation. The probability for a motion response reached 100% at 0.21 mW/mm^2^ (Fig. 1h). In summary, optimal zTrpa1b activation was achieved using violet light with an intensity of 0.21 mW/mm^2^ and with a duration of 30 ms.

### Exogenous expression of zTrpa1b enables light dependent neuronal activation

**Figure 2:**
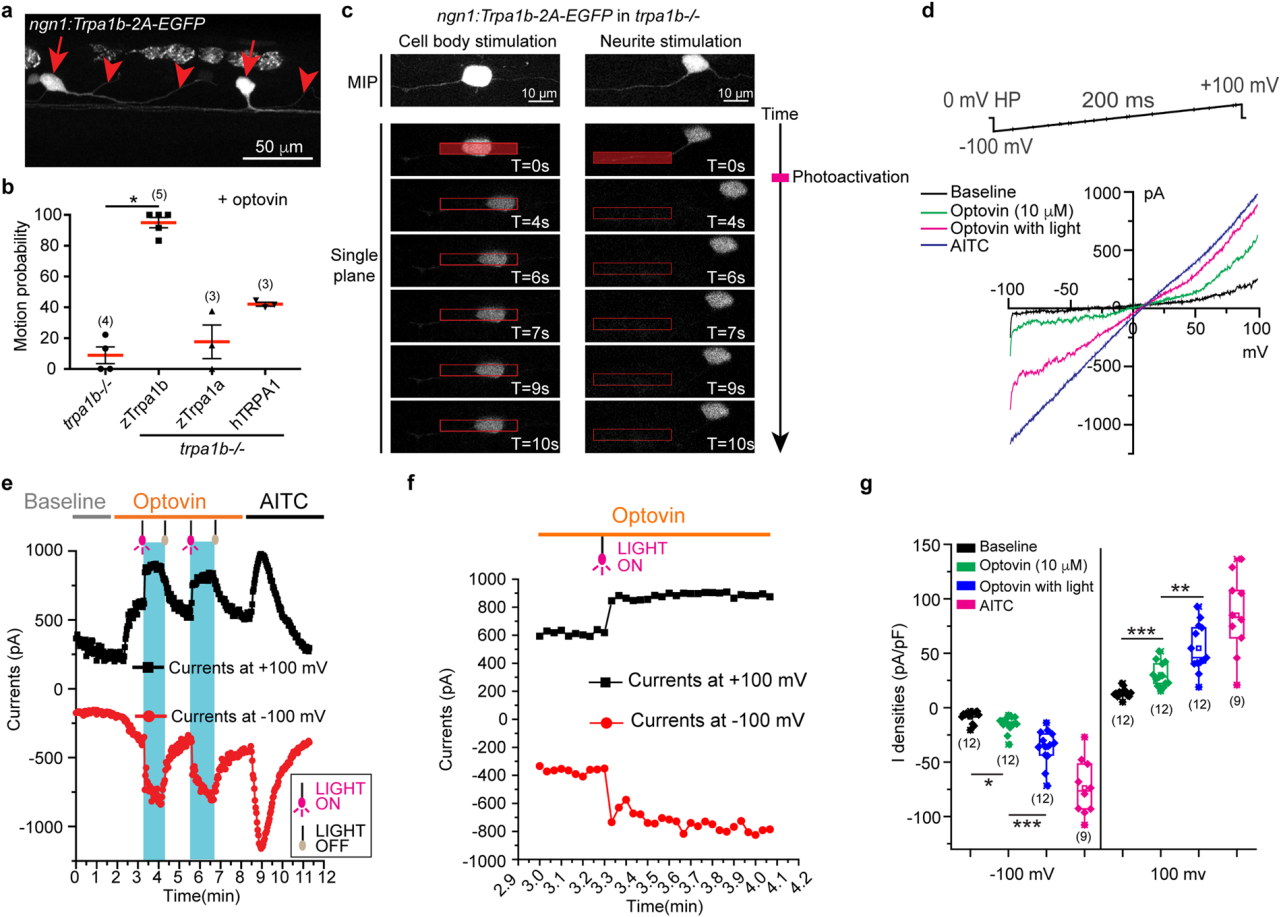
Exogenous expression of zTrpa1b in sensory neuron of *trpa1b*−/− mutants and sub-cellular photo-activation. (**a**) Maximum intensity projection showing mosaic expression of zTrpa1b in Rohon-Beard neurons in the trunk of a zebrafish larva at 2.5 dpf. Red arrows and arrowheads indicate the Rohon-Beard neuronal cell body and its neurite projection, respectively. (**b**) Photomotor response was tested in *trpa1b* mutant (*trpa1b*−/−) controls, or in *trpa1b* mutants expressing zebrafish Trpa1b (zTrpa1b), zebrafish Trpa1a (zTrpa1a) or human TRPA1 (hTRPA1) in Rohon-Beard neurons (Video S1), pretreated with 10 µM optovin. Values are means ± SEM from more than 3 experiments. Each experiment has n > 10 per condition. *p < 0.05. (**c**) Subcellular photo-activation of a zTrpa1b expressing Rohon-Beard neuron in a *trpa1b* mutant. MIP, confocal maximum intensity projection. Representative single plane time series images with photo-activation targeting neuron cell body (n = 10) (left panel; Video S2) or neurite (n = 9) (right panel; Video S3) in the trunk region of a zebrafish larvae *in vivo*. Red rectangular box indicates the time and location where photo-activation was made. (**d**) Current-Voltage relationships of zTrpa1b currents without treatment (black), 10 μM Optovin alone (green), 10 μM Optovin and light (magenta) and AITC (blue) (**e**) Peak whole cell zTrpa1b currents measured at −100 mV (black trace) and +100 mV (red trace); Optovin-dependent photocurrents are highlighted in blue boxes, bounded by light switch-on (magenta bulb) and light switch-off (gray bulb). (**f**) Data from (**e**) with higher time resolution. (**g**) Box chart showing peak current densities of zTrpa1b in indicated conditions at −100 mV and +100 mV (n = />9, error bars represent SEM).

In order to test if exogenous expression zTrpa1b is sufficient for optovin/light driven photoactivation, we expressed zTrpa1b mosaically in a subset of Rohon-Beard sensory neurons of *trpa1b* mutant zebrafish using the *neurogenin1* (*ngn1*) promoter^39,40^ In wild-type early zebrafish larvae, *trpa1b* is expressed broadly in Rohon-Beard neurons and a subset of trigeminal neurons^41^. The *ngn1* promoter drives expression in Rohon-Beard neurons and dorsal root ganglia among other tissues distributed in the head region^39^ (Fig. 2a). Mosaic re-introduction of zTrpa1b, using the *ngn1* promoter, in *trpa1b* mutants restored zebrafish motor response when the whole larvae were stimulated with light in the presence of optovin (Fig. 2b). This motion response is consistent with a similar manipulation where ChR2 is expressed in somatosensory neurons in zebrafish, which is sufficient to induce escape behavior in zebrafish upon light activation^42^; or with the expression of a chemical optogenetic system (LiGluR) in zebrafish Rohon-Beard neurons to optically control movement^43^. Our results show that exogenous expression of zTrpa1b in sensory neurons can enable optovin/light driven photomotor response in *trpa1b* mutant.

There are two Trpa1 orthologs in zebrafish, zTrpa1a and zTrpa1b. Unlike *trpa1b*, *trpa1a* expression is restricted to a few cells in the most posterior vagal sensory ganglion in the head of the larvae^41^. As zTrpa1a and zTrpa1b share 59% protein identity, we tested if Trpa1a is also sensitive to optovin activation. When zTrpa1a was expressed in the Rohon-Beard neurons of *trpa1b* mutants, there was no rescue of optovin/light activity (*p* = 0.5714 versus *p* = 0.0159 for zTrpa1b expression) (Fig. 2b, Video S1). This suggests that zebrafish Trpa1b but not Trpa1a is sensitive to optovin/light activation. Human TRPA1 (hTRPA1) and zebrafish Trpa1b share 46% protein identity. Our previous data suggested that hTRPA1 is sensitive to optovin^37^. In order to compare the activity of optovin on hTRPA1 versus zTrpa1b, we transiently expressed hTRPA1 in Rohon-Beard neurons and performed a light activation assay. We found that *trpa1b* mutants expressing hTRPA1 were less sensitive to optovin/light activation compared to mutants expressing zTrpa1b (*p* = 0.0571) (Fig. 2b). Overall, our results suggest that optovin/light triggers gating of zebrafish Trpa1b but not Trpa1a. Optovin also has some activity on human TRPA1, but to a lesser extent as compared to zebrafish Trpa1b.

### zTrpa1b/optovin photoactivation is highly sensitive in neurons

To further examine the sensitivity of optovin-induced neuronal cell activation, we performed sub-cellular photoactivation on zTrpa1b expressing Rohon-Beard neurons in *trpa1b* mutant larvae. The *ngn1* promoter was used to drive transient, mosaic transgene expression, and coexpression of GFP enabled visual identification of neurons expressing zTrpa1b. When either the cell body (Fig. 2c; Video S2) or the neurite region (Fig. 2c; Video S3) of a single Rohon-Beard neuron was photo-activated using a laser scanning confocal microscope setup, movement in the zebrafish larvae was triggered. These results suggest that zTrpa1b/optovin is robust in provoking light-induced neuron activation.

### Kinetics of zTrpa1b/optovin-dependent photocurrents

We recorded the channel activity of zTrpa1b in a heterologous system (expressed in 293 T cells) using whole cell patch clamp recordings. In similarity to hTRPA1, zTrpa1b elicited robust currents in response to AITC with a typical near-linear current-voltage (IV) relationship and a reversal potential of ~5 mV (Fig. 2d). Optovin alone elicited higher basal currents but at resting membrane potentials of physiological significance to most cells (negative of −50 mV), zTrpa1b/optovin-dependent photocurrents displayed a substantial gain in response to light stimulation. To characterize the stability and kinetics of the zTrpa1b/optovin-dependent photocurrents, we recorded these photocurrents using brief pulses of light. The light-induced activation was very rapid, with response latency less than the 2 seconds acquisition interval (Fig. 2e,f). This was consistent with the biological data where zebrafish larvae exhibited motion response to light activation after a millisecond scale delay (Fig. 1g). The photocurrents were sustained when the light was on, indicating that light-dependent activation does not result in rapid inactivation of zTrpa1b. When the light was switched off, the zTrpa1b/optovin photocurrents decayed relatively slowly, with a deactivation time constant τ_off_ of 11.4 s ± 1.3 s at −70 mV. Importantly, the light activation of optovin-primed zTrpa1b was reversible and repeatable, as additional light exposures induced similar photocurrents (Fig. 2e). The zTrpa1b/optovin photocurrent densities during these repeated light pulses were only modestly lower than those elicited by the AITC-activated zTrpa1b channels (Fig. 2g).

### Photo-activity of optovin analogs

**Figure 3:**
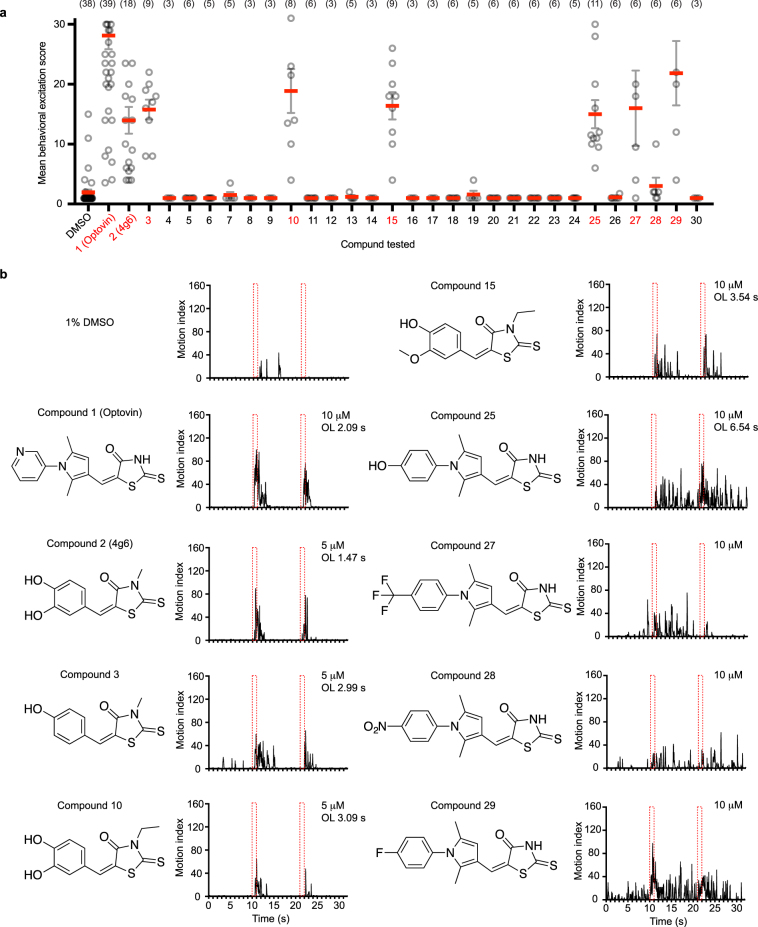
Optovin structure-activity relationship analysis. (**a**) Photomotor response assay (PMR) on WT larvae at 3 dpf pre-treated with DMSO control or the indicated compound for 1 h. Mean behavioral excitation score was calculated by taking the 75^th^ percentile of the motion index from 1 s–3 s following the light stimulus. Data are the mean value from at least three experiments. Each experiment contained 4 larvae. A subset of derivatives showed a higher than baseline motion activity and are highlighted in red. The chemical structure of the compounds is shown in S2. (**b**) PMR assay results of the subset of compounds indicated in (**a**) as red. Motion of the larvae is plotted again time. The red dotted rectangles indicate the timing of 1 s light pulses. OL, off latency.

The optovin chemical structure contains three rings: a pyridine, a pyrrole and a rhodanine ring (S2 compound 1). We have previously identified several active analogs of optovin showing different durations of effect in the PMR assay^37^. These results suggest that optovin’s biological activity depends on specific structural features that can be fine-tuned for shorter or longer lasting effects. Here, we further modified optovin by adding a benzene ring to the different ring structures (S2 compound 20–24); adding either an electron donating group (S2 compound 25, 26) or an electron accepting group (S2 compound 27–30) to the pyridine ring. We also tested modifications to the previously identified active analog 4g6 (S2 compound 2–19). We found that compounds 1, 2, 3, 10, 15 and 25 showed a light dependent motion inducing activity on the PMR assay performed on WT zebrafish at 3 dpf, while compound 27, 28 and 29 showed non-light specific increase in motion (Fig. 3a,b). Compound 1 (optovin) showed the most robust light dependent motion response with a motion off latency of ~2 s. On the other hand, compound 2 (4g6) and compound 25 showed moderate light dependent motion response but with a shorter off latency of ~1.5 s and a longer off latency of ~6.54 s, respectively (Fig. 3b). Compounds 27, 28 and 29 appeared to induce an increase in basal motion in larvae (Fig. 3b).

**Figure 4:**
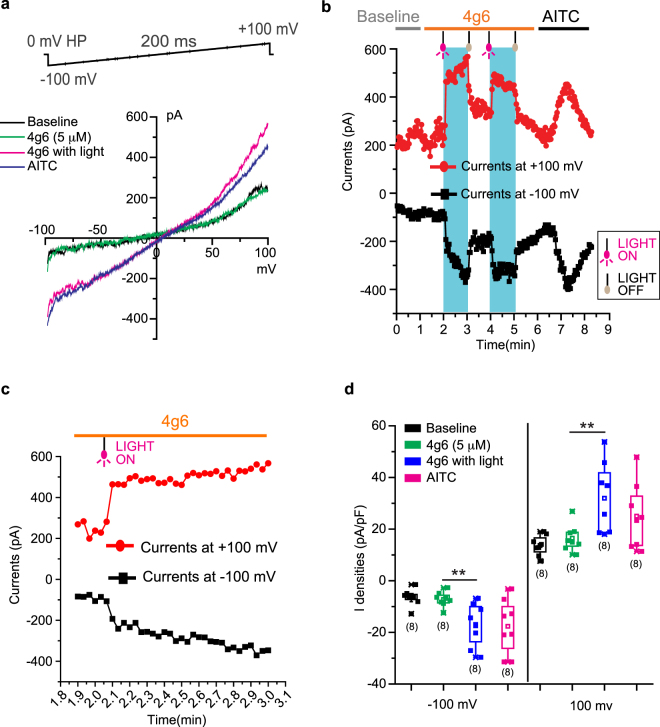
Electrophysiology analyses of 4g6. (**a**) Current-Voltage relationships of zTrpa1b currents without treatment (black), 5 μM 4g6 alone (green), 5 μM 4g6 and light (magenta) and AITC (blue). (**b**) Peak whole cell zTrpa1b currents measured at −100 mV (black trace) and +100 mV (red trace); 4g6-dependent photocurrents are highlighted in blue boxes, bounded by light switch-on (magenta bulb) and light switch-off (gray bulb). (**c**) Data from (**b**) with higher time resolution. (**d**) Box chart showing peak current densities of zTrpa1b in indicated conditions (n = 8, error bars represent SEM).

Using a whole cell patch clamp recording configuration identical to that used for optovin, we characterized the photocurrents elicited by the zTrpa1b/4g6 combination. Upon light stimulation, 4g6-primed zTrpa1b generated robust photocurrents that were indistinguishable in their amplitude and IV characteristics to AITC-activated currents (Fig. 4a). Interestingly, in contrast to optovin, the treatment of cells with 4g6 alone (in absence of light) did not result in a measurable gain in zTrpa1b currents. In this regard, 4g6 will be more suitable than optovin for applications where minimal basal current is critical. The light-induced activation of zTrpa1b/4g6 was instantaneous, the photocurrents were sustained as long as the light was switched on (Fig. 4b,c), and the light-induced activation was reversible and repeatable (Fig. 4b). After the light was switched off, the zTrpa1b/4g6 photocurrents decayed much more rapidly (τ_off_ = 7.1 ± 1.1 s at −70 mV) than zTrpa1b/optovin photocurrents (τ_off_ = 11.4 ± 1.3 s at −70 mV) indicating a ~40% decrease in off-rate. These measurements correlate well with *in vivo* observations that 4g6 had a shorter off motion latency than optovin (Fig. 3b) and this shorter off latency should prove beneficial for many other biological applications. Notably, the decreased off-rates of zTrpa1b/4g6 photocurrents do not come at the expense of current density; these photocurrents in fact display current densities comparable to AITC-activated zTrpa1b channels (Fig. 4d). We speculate that the difference in the chemical structure between optovin and 4g6 accounts for the difference in the τ_off_ value. Overall, our data suggest that with the use of different photo-activated ligands for zTrpa1b, the channel off latency can be adjusted and modified in an iterative manner for the specific biological application.

### zTrpa1b/optovin allows cellular activation in non-neuronal cells

**Figure 5:**
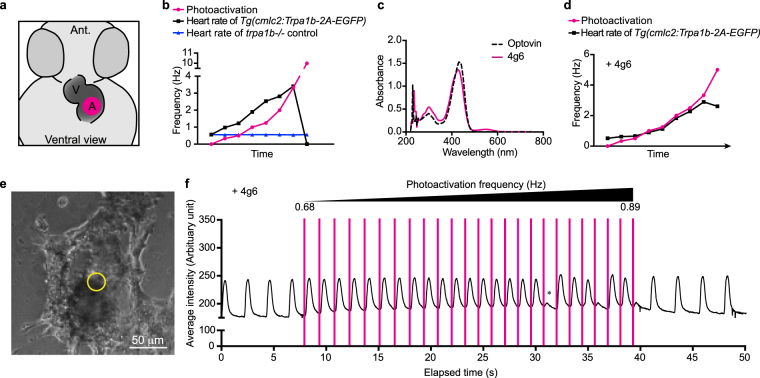
Optogenetic pacing of zebrafish hearts *in vivo* and human stem cell-derived cardiomyocytes *in vitro*. (**a**) Diagram of zebrafish larval heart. Magenta circle indicates the location of photo-activation. V, ventricle; A, atrium; Ant, anterior. (**b**) Heart pacing experiment on *trpa1b*−/− larvae expressing zTrpa1b in the cardiomyocytes at 2 dpf. Heart pacing was performed by photo-activating the atrium of the heart *in vivo* with violet light. Representative graphs show the change in heart rate for *Tg*(*cmlc:Trpa1b-2A-EGFP*) (black line, n = 11, Video S4) or *trpa1b*−/− control (blue line, n = 7) and the corresponding photo-activation frequency (magenta line) with 10 μM optovin. (**c**) Absorbance of optovin and 4g6 at the indicated wavelengths. (**d**) Similar heart pacing experiment as in (**e**) with 10 μM 4g6 treatment (Video S5; n = 8). (**e**) Brightfield image of zTRPA1b-expressing human stem cell-derived cardiomyocyte with sub-region indicated for the trace in (**f**). (**f**) Mean pixel intensity over time as an index of local contractile-displacement in the presence of 4g6. Photoactivation light pulses are indicated with magenta bar. Asterisk indicates a loss of 1:1 pacing-capture at a high photo-activation frequency (n = 3).

Because zTrpa1b is normally expressed in neurons, we wanted to test whether or not zTrpa1b/optovin/light could be use in non-neuronal contexts. The feasibility of optogenetic control of cardiac function *in vivo* has been demonstrated previously with the expression of halorhodopsin and channelrhodopsin^44^ or with a photoswitchable affinity label that specifically targets endogenous K^+^ channels^14^. We performed a heart pacing experiment in which zTrpa1b was expressed in cardiomyocytes of zebrafish larvae using the *cmlc2* promoter^45^ followed by optogenetic heart pacing experiments. Since a strong endogenous heartbeat could potentially interfere with the ability of the cardiomyocytes to follow external stimulations, we injected ivabradine to selectively inhibit endogenous pacemaker activity. We then performed heart pacing by applying pulses of violet light to the atrium of the zebrafish heart at various frequencies *in vivo* (Fig. 5a). We found there was no significant difference in heart rate between embryos treated with DMSO control or optovin in the dark (S3), but in the presence of optovin and a low frequency violet light pulse, the heart beat approximately twice as fast as the driving light pulse (Fig. 5b; Video S4). At a higher stimulation frequency of 3 Hz, the heart entered a tetanic state, suggesting that cardiomyocytes were calcium overloaded, exceeding the cellular calcium re-uptake mechanisms. *trpa1b* mutant, on the other hand, showed no correlation between the heart rate and the photoactivation frequencies (Fig. 5b). We hypothesized that the slow off rate of optovin-induced zTrpa1b activation led to sustained cation influx, leading both to a faster beat rate and subsequent tetany. We therefore decided to repeat the pacing experiments using 4g6, which has an optical absorbance spectrum very similar to optovin (Fig. 5c) but a faster inactivation-rate. Using the same experiment setup, we were able to observe a 1:1 ratio of heart rate to pacing flashes, which was maintained across a range of driving frequencies (Fig. 5d; Video S5). These results suggest that the shorter inactivation rate characteristic of 4g6 is required for faithful 1:1 cardiac pacing. Since cardiomyocytes do not express zTrpa1b endogenously, these results suggest that no additional neuron-specific component is required for optovin induced cellular activation, even in non-neuronal cells. Exogenous expression of zTrpa1b in cardiomyocytes allows artificial pacing of the heart *in vivo*.

### zTrpa1b combined with 4g6 allows for pacing of human stem cell-derived cardiomyocytes

We next tested if this zTrpa1b chemo-optogenetic tool could be used in other vertebrate systems apart from zebrafish. We transduced human stem cell-derived cardiomyocytes by lenti-virus to drive zebrafish Trpa1b expression. We then performed pacing experiments similar to the *in vivo* heart pacing experiments performed in zebrafish larvae (Fig. 5e). Expression of zebrafish Trpa1b did not affect the ability of cardiomyocytes to beat spontaneously (Fig. 5f). Cardiomyocyte pacing was achieved by pulses of violet light with increasing frequency in the presence of 4g6. zTrpa1b combined with 4g6 allowed for pacing of cardiomyocytes at the frequency range between 0.68 Hz to 0.85 Hz, after which the cardiomyocytes lost the 1:1 stimulation to beating ratio (Fig. 5f). The cardiomyocytes returned to spontaneous beating after photoactivation was stopped. Together, these results suggest that the zTrpa1b/ligand chemo-optogenetic actuator is compatible with mammalian cell systems and allow tight control of cell activation in zTrpa1b expressing cells using violet light. As has been noted elsewhere, there is a need for high-throughput systems compatible with optical pacing and optical assessment of cardiac cell function for new drug development^46^. zTrpa1b/4g6 could be applied as a scalable stimulation setup for optical pacing in human stem cells.

Overall we show that zTrpa1b, coupled with a photo-activated channel ligand, as a robust violet light activated chemo-optogenetic actuator. The channel off rate of zTrpa1b can be adjusted with the use of different photo-activated channel ligands. This zTrpa1b and photo-activated ligand pairing allows tight spatial and temporal control of the activation of excitable cells.

## Discussion

Although others have successfully achieved high conductance and calcium permeability with a variety of chemical optogenetic approaches^32,47,48^, we believe the TRPA1/ligand chemo-optogenetic actuator described here offers a unique combination of characteristics and potential advantages. For example, TRPA1 has a high conductance of about 100 pS^49^ while the conductance of a single ChR2 is below 1 pS^22,23^. As a result, zTrpa1b may enable weaker promoters to be used to drive channel expression while still achieving sufficient light-induced activation of cellular activity. TRPA1 has a slight selectivity for Ca^2+^ versus Na^+^^50–52^. TRPA1/ligand can potentially be employed as a Ca^2+^ mobilization tool for activation of downstream signaling, even in non-excitable cells. In addition, TRPA1/ligand has tunable kinetics. While the light-induced activation of zTrpa1b is in the rapid millisecond range *in vivo*, the channel off latency of optovin and its derivatives differ, allowing for flexibility in accommodating applications with different channel off-rate requirements even in the same genetic system. These differences in the rate of inactivation may parallel the wide range of recovery timescales observed for the light-induced coupling of flavins and cysteines in LOV domain proteins^53^. Several factors might explain why the structural differences between optovin and 4g6 influence the rate of inactivation. Perhaps structural differences affect solvent access to the active site, thereby influencing rate of return to the ground state. Similarly, the two compounds may bind to Trpa1b in slightly different positions based on their different geometries, and this difference may influence the rate of the decoupling reaction. Alternatively, electronic differences inherent to optovin and 4g6 themselves (e.g. pyrrole versus catechol) may influence the rate of the decoupling reaction.

zTrpa1b and ligands/light pairing can be used for prolonged cell activation, in the minutes range, while remaining sensitive to repeated photo-activation. Optovin has a maximum spectral response at approximately 430 nm and achieves 100% of the maximum behavioral response with violet light power as low as 0.21 mW/mm^2^ (Fig. 1h), which is comparable with ChR2 at 0.5 mW/mm^2^ blue light^54^. In theory, zTrpa1b and optovin pairing is well suited to be used in conjunction with red-shifted opsins such as Chrimson^10^, although experimental validation is needed. This would allow for a substantial separation of activation wavelengths as well as a low excitation light power.

In addition, zTrpa1b can be expressed, properly localized, and functional across several different cell types and different animal species. The system exhibits minimal-to-no basal current in the absence of light with 4g6. Interestingly, optovin does cause some basal current prior to photoactivation (Fig. 2f). It is possible that optovin has some basal reactivity, whereas 4g6 does not (Fig. 4c). It is noteworthy that the basal current with optovin seems most pronounced at high, positive voltages, and is minimal at more physiologically relevant negative currents. The observation that addition of optovin in the dark or even under dim ambient light conditions does not trigger movement in WT embryos suggests that the basal current induced by optovin alone is perhaps biologically negligible. Furthermore, photo-activated chemical ligand is required for light activation of zTrpa1b channels. This ligand requirement for photo-activation of channels can provide additional spatial and temporal control for light-induced activation. For example, transgenic animals do not need to be kept in special light conditions to prevent unintended light-induced activation of channels.

There are distinct attributes of this system that might be disadvantageous. The deactivation time constant τ_off_ for the short off rate ligand 4g6 is approximately 7.1 s ± 1.1 s, which is relatively long compared to other photo-activated opsins (typically in the millisecond range), but shorter than the bistable/step-function opsins (in the minute range without light-induced inactivation)^55^. Cautions should be made in applications where rapid deactivation is needed. It should be noted that this τ_off_ value was measured in ectopically transfected HEK293T cells, which could account for the slower deactivation time relative to what is observed *in vivo*. It is also important to note that this zTrpa1b and ligand/light pairing consists of two exogenous components, a gene product and a photoactivatable ligand, requiring the addition of the small molecule to the experimental set up. Delivering a photochemical to tissues can sometimes be problematic or toxic. Fortunately, for this system to be functional, a relatively low ligand concentration (in micromolar range) is needed, and the system becomes active with very short incubation times. While it is possible exogenous agonists or cell messengers can activate the over-expressed Trpa1b, we have not observed any endogenous activation of the overexpressed Trpa1b channel. Transgenic fish with Trpa1b overexpressed specifically in the heart or in the Rohon-Beard neurons show normal development and are fertile.

We anticipate future optimization efforts in the following directions:

Identifying photoactivatable ligands with diverse absorbance profiles: In searching for photoactivatable ligands that confer a different channel off latency profile, we have identified several optovin analogs that retain light sensitivity (Fig. 2; compound 3, 10, 15 and 25). We envision future efforts in the modification of ligands with the addition of conjugate groups to increase the ligand absorbance. This will result in an increased excitation wavelength for zTrpa1b photoactivation, offering unique adaptability options where optogenetic actuators can be combined to achieve different spectral requirements.

Modification of channel selectivity: Using a structure-informed electrostatic model for pore selectivity, variants of channelrhodopsin have been engineered to become chloride selective^8,9,12^. The human TRPA1 has a tetrameric assembly profile^56^ and its protein structure has recently been partially resolved^57^. Similar principles used for channelrhodopsin could be applied to TRPA1 in order to create a high conductance anion channel.

Endogenous TRPA1 channel activation: Zebrafish Trpa1b and human TRPA1 share 46% protein identity. Our current and previous analyses^37^ indicate that human TRPA1 is also sensitive to optovin. However, optovin appears to be more effective in activating the zebrafish Trpa1b than the human TRPA1 (Fig. 2b). Further optimization of optovin may be required to identify other species-specific TRPA1 photo-activated ligands.

## Methods

### Zebrafish

Zebrafish (*Danio* rerio) wild-type TuAB or *trpa1b* mutants^41^ were used. Animals were maintained and embryos were obtained according to protocols approved by the Massachusetts General Hospital Institutional Animal Care and Use Committee.

### DNA constructs and injection

DNA expression constructs were made using the Multisite Gateway Cloning System (Invitrogen) into Tol2 vectors^58^. Gateway BP- and LR- reactions were carried out according to manufacturer’s instructions (Thermo Fisher Scientific). Overlapping PCR was performed to add a 2A-mCherry or 2A-EGFP sequence to the C-terminus of zebrafish Trpa1a (zTrpa1a; EU826643), Trpa1b (zTrpa1b; EU826642) or human TRPA1 (hTRPA1; NM_007332) to create a Gateway middle entry clone via a Gateway BP reaction. The middle entry constructs were then combined with the p5′E entry vector containing the *cis-*regulatory element of the *neurogenin1* gene (-*3*.*1ngn1*) for Rohon-Beard neuron expression^39,40^, or 0.85Kb of the cardiac myosin light chain promoter for cardiomyocyte expression^45^, together with destination vector *pDestTol2pA2* in an LR reaction to generate the final expression construct. Expression of the construct was obtained by injecting 3 nL of a solution containing 6 ng/µL of DNA plasmid and 6.5 ng/µL *in vitro* transcribed (Ambion) Tol2 transposase mRNA into the cytoplasm of one-cell stage *trpa1b* mutant embryos.

### Compound synthesis

Compounds 11, 17 and 19 were synthesized with this general procedure: In a 100 mL round bottom flask, equimolar amounts of corresponding rhodanine or hydantoin (10 mM), anhydrous sodium acetate (10 mM) were added in glacial acetic acid (10 mL) and then the corresponding aromatic aldehydes (10 mM) were added to the reaction mixture. The mixture was stirred under reflux condition for 4–8 h. The progress of the reaction was monitored by TLC (20% ethyl acetate: hexanes). After completion of the reaction, the reaction mixture was poured into ice-cold water. The precipitate was filtered off and washed with water (3 * 15 mL), dried, and purified by recrystallization in ethanol as solvent to give 80–85% yield. Detailed synthesis pathways are shown in S1.

### Electrophysiology

HEK293T cells were transient expressed with 3 µg of TRPA1 plasmid (*pCMV-zTrpa1b-FLAG*) and 0.5 µg of *GFP* per well, in a 6-well plate, for 24 h. After 24 h the cells were plated on Poly-D- Lysine coated 12 mm cover slips. The cover slips were transferred to a recording chamber containing extracellular solution composed of (in mM): 140 NaCl, 5 KCl, 3 MgCl_2_, 10 D-glucose, 10 HEPES; pH 7.4 adjusted with NaOH. The internal solution contained (in mM): 122 Cesium methanesulfonate, 1.8 MgCl_2_, 9 EGTA, 14 creatine phosphate (Na-salt), 4 MgATP, 0.3 NaGTP, 10 HEPES, pH 7.2 adjusted with CsOH. The internal solution was filled in borosilicate glass pipette freshly pulled and fire-polished to a resistance of 3–5 MΩ. Whole cell currents were measured using Axopatch 200B (Molecular Devices) and pClamp data acquisition software using a current-voltage ramp protocol (applied from −100 to +100 mV in 200 ms, every 2 s and at holding potential of 0 mV). The acquired data were digitized using Digidata1440A (Molecular Devices). Currents were filtered at 5 kHz (lowpass, Bessel) and sampled at 10 kHz prior to analysis with Clampfit software (Molecular Devices) and Originpro 9.1 (OriginLab). For measuring photocurrents, cells were patched in whole cell configuration and a ramp protocol was applied after break-in. After perfusion of bath solution with or without photo-activated ligand (>1 min), Violet/blue light (434/17 nm, 0.8 mW/mm^2^) was switched on for indicated times. The illumination source was Lambda DG4 (Sutter Instruments) containing Xenon Arc bulb and the light was filtered using 434/17 nm BrightLine Bandpass filter (Semrock, NY, USA). The light switches by DG4 were timed and controlled using Slidebook control and acquisition software (3i). All experiments were done at RT. The p values were calculated using two sample student t-test with equal variance assumed. The time constants (τ values) were derived by fitting the current-time (IT) traces, obtained at −100 mV or −70 mV, to a one-phase exponential decay function with a time-constant parameter using OriginPro 9.1 software.

### Absorbance measurement

Compounds were dissolved in DMSO at 500 µM and absorbance (UV-Vis) was measured using a NanoDrop 1000 (Thermo Scientific).

### Zebrafish heart pacing

2 dpf Tg(*cmlc2:zTrpa1b-2A-EGFP*) zebrafish larvae were pre-treated with 10 µM optovin or 10 µM 4g6 for 1 h before the pacing experiment. In order to reduce the intrinsically high heartbeat of zebrafish larvae, a potential source of interference, 2 nL of 20 mM Ivabradine (E147, AK Scientific, Inc.) was micro-injected directly into to space of the larvae, immediately prior to the pacing experiment. The method of pericardial space injection in zebrafish has been previously described^59^. Schott longpass absorption glass (RG610, Chroma) was placed between the injection mold and the bright-field light source during injection to reduce unwanted photoactivation. Larvae were then embedded in 1% low melting agarose with their ventral side down in a glass bottom petri dish. The photoactivation setup was similar to that used for the PMR assay shown in Fig. 1f and g, except an NA 1.2 40x water immersion objective was used. The Area of photoactivation was restricted with the aperture diaphragm to a circular area of ~1260 μm^2^. Photo-activation was made with a pulse of 30 ms and a wavelength of 415 nm light at 5–10% output power (1.3 mW/mm^2^–2.9 mW/mm^2^) directed at the atrium of the heart. Experiments were conducted at room temperature. Frequency of heartbeat was calculated by averaging the time for four cycles of atrial contraction. Bright field time-lapse was captured at ~100 FPS.

### Cardiomyocyte pacing

Human cardiomyocytes were differentiated from pluripotent stem cells, dissociated, and re-plated within a 24-well dish as previously described^60^. *pLenti-CMV-zTrpa1b-2A-mCherry* was constructed using the *pLenti-CMV-Puro-Dest* backbone (Addgene plasmid 17452)^61^. Lentiviral particles were produced and used to transduce cardiomyocytes as previously described^60^. Cardiomyocytes were imaged in maintenance media supplemented with 5 µM 4g6 on a Nikon Eclipse Ti-U inverted microscope fitted with an environmental control enclosure (InVivo Scientific: CH.IVS.100) to maintain temperature, humidity, and carbon dioxide. Images were acquired under red-passed (Chroma: HQ615lp) brightfield illumination using an Evolve 128 (Photometrics) camera at 100 frames/sec. Excitatory 415 nm light pulses of 50 ms duration at 50% maximum output were generated from a dedicated LED light source (BLS-LCS-0415-03-22; Mightex) and delivered by light-guide to the microscope’s fluorescence excitation port and then to the cells using a 10/90 beam splitter (Chroma: 21012) filter block.

### Statistical Analyses

All results are expressed as means ± SEM. A two-tailed unpaired student’s t test with Mann-Whitney post-test was used to determine *p* values. The criterion for statistical significance was *p* < 0.05. Statistical analysis was performed using Prism (GraphPad Software).

The datasets generated and/or analyzed during the current study are available from the corresponding author on reasonable request.

## Electronic supplementary material

Supplementary Information

Video 1

Video 2

Video 3

Video 4

Video 5
