# Supplementary material for: A high-conductance chemo-optogenetic system based on the vertebrate channel Trpa1b
Source: Sci Rep. 2017 Sep 19;7:11839. doi: 10.1038/s41598-017-11791-z (PMC5605526; doi:10.1038/s41598-017-11791-z)
Supplement: Supplementary file 1 — Supplementary Information [file 41598_2017_11791_MOESM1_ESM.pdf]

Title

A high-conductance chemo-optogenetic system based on the vertebrate channel Trpa1b

Authors

Pui-Ying Lam[1,2,3,4,*], Suresh K. Mendu[5], Robert W. Mills[1], Baohui Zheng[1], Hugo Padilla[1], David J. Milan[1], Bimal N. Desai[5] and Randall T. Peterson[1,2,3,4, *]

[1] Cardiovascular Research Center, Department of Medicine, Massachusetts General Hospital, Harvard Medical School, Charlestown, MA 02129, USA
[2] Department of Systems Biology, Harvard Medical School, Boston, MA 02115, USA
[3] Broad Institute, Cambridge, MA 02142, USA
[4] Department of Pharmacology and Toxicology, College of Pharmacy, University of Utah, Salt Lake City, UT 84112, USA
[5] Department of Pharmacology, University of Virginia School of Medicine, Charlottesville, VA 22908, USA
* Correspondence to: Pui-Ying Lam, email: PuiYing.Lam@pharm.utah.edu
* Correspondence to Randall T. Peterson, email: randall.peterson@pharm.utah.edu

Supplemental Methods

Photomotor response (PMR) assay

For Fig. 1a-e and Fig. 3, Zebrabox (ViewPoint Behavior Technology) with the built in Zebralab quantification program was used. Four 3 dpf larvae in 300 μL volume were placed in individual wells of a square flat-bottom 96-well plate (7701-1651; Whatman UNIPLATE). Light flashes were programed as: 10 s no light, 1 s 100% light, 10 s no light, 1s 100% light and 10 s no light. All PMR experiments were conducted at room temperature. Mean behavioral excitation score is calculated by taking the 75th percentile of the motion index from 1 s - 3 s following the light stimulus. To calculate the off latency, five consecutive data points at a 30 ms interval were first combined and then averaged. The time point where the last motion index at the value 15 was calculated as the off latency. The value of 15 is the lowest value that allows us to detect above background motion activity. Light intensity was measured using a hand-held laser meter (LaserCheck, Coherent 1098293).

For Fig. 1f - h, experiments were performed on WT larvae at 3 dpf using an inverted compound microscope (AxioObserver A1; Zeiss) equipped with an EMCCD camera (C9100; Hamamatsu), a violet LED light source (415 nm) with a CW 310 mW maximum output power (BLS-LCS-0415-03-22; Mightex) which is controlled by a BioLED light source control module (BLS-SA02-US; Mightex) and a pulse master multi-channel stimulator (A30; World Precision Instruments). Schott longpass absorption glass (RG610; Chroma) was added in the transmitted light path to eliminate unwanted excitation. A dichroic mirror (T510LPXRXT; Chroma) is used for appropriate excitation of optovin and bright field acquisition. The software MetaMorph (Molecular Devices) was used to control the execution of TTL signals and camera capture using the stream acquisition with trigger function. Larvae were individually placed in 100 μL of media in a single well of a 96-well clear bottom black microplate (07-200-567; Fisher Scientific). A NA 0.25 5x objective was used. Photo-activation was made with a pulse of 30 ms 415 nm light in the mid-trunk region of zebrafish larvae. Bright field time-lapse was captured for 500 frames at ~103 frames per second (FPS). A light pulse was applied at frame 20. Percentage responding was quantified based on whether there was any motion in the entire acquisition period. Response time is the duration from the beginning of the light pulse to the first movement of the larvae.

For Fig. 2b, the corresponding construct was injected in *trpa1b* mutant embryos as described previously. At 2 dpf, larvae with one or more Rohon-Beard neurons expressing the transgene were picked and treated with 10 μM optovin for 1 hour. The experimental setup previously described for Fig. 1f – h was used.

For Fig. 2c, similar to the experimental setup used for Fig. 2b, *trpa1b* mutant larvae were injected with the *ngn1:zTrpa1b-2A-EGFP* construct and screened for mosaic Rohon-beard neuron expression at 2 dpf. Larvae were incubated with 10 μM optovin for 1 h before experiment. Larvae were then briefly anesthetized with

0.02% MS222 before being mounted laterally in 1% low melting agarose. The mounting protocol has been previously described [1]. Larvae were than incubated in E3 without MS222 before the photo-stimulation experiment. Photoactivation was performed on a confocal microscope (Zeiss Observer Z1) using a NA 1.2 40x water immersion objective with the bleaching function in the Zen Black 2009 (Zeiss) software. A 405 nm laser in line scan mode at 80% power was used to activate a 40.5 μm x 5.7 μm rectangular region for approximately 4 s. Time-lapse confocal single plane images were acquired before and after photoactivation was performed.

Ordering information
1 (optovin; 4901; Tocris), 2 (4g6; Amb2633086; Ambinter), 3 (F1284-0264; Life Chemicals), 4 (Z221260068; Enamine), 5 (5886745; ChemBridge), 6 (5899736 ; ChemBridge), 7 (5886249; ChemBridge), 8 (S762571; Sigma), 9 (5734846; ChemBridge),10 (5587606 ; ChemBridge), 12 (STOCK5S-87834; InterBioScreen), 13 (5378191; ChemBridge), 14 (6633951; ChemBridge), 15 (5161542; ChemBridge), 16 (Z44297155; Enamine),18 (OSSK_509906 ; Princeton BioMolecular Research), 20 (7016256; ChemBridge), 21 (AKOS005128847; Akos), 22 (2329-0315; ChemDiv), 23 (6193919; ChemBridge), 24 (4490-1974; ChemDiv), 25 (STOCK5S-39621; InterBioScreen), 26 (6024796; ChemBridge), 27 (6315437; ChemBridge), 28 (5912985; ChemBridge), 29 (6020024; ChemBridge) and 30 (5908419, ChemBridge).

SI

S1. Chemical synthesis information of compound 11, 17 and 19.

S2. The chemical structure of optovin and its analogs.
Chemical structure of optovin (1) and its derivatives (2-30) used in Figure 3a. Compound 1, 2, 3, 10, 15 and 25 showed light-evoked activity in the photomotor response assay performed on WT zebrafish at 3 dpf.

S3. Heart rate of *Tg(cmlc2:zTrpa1b-2A-EGFP)* zebrafish heart at 2 dpf. Larva was pretreated with DMSO or 10 μM optovin and heart rate was measured in dark. BPM, beats per minute. ns, not significant.

Video S1. Photomoter response of *trpa1b-/-* mutants with transient, mosaic expression of zebrafish Trpa1b or Trpa1a in Rohon-Beard neurons using the *neurogenin1* (*ngn1*) promoter. Experiments were performed at 2.5 dpf. Larvae were pretreated with 10 μM optovin.

Video S2. Time-lapse images of a zTrpa1b expressing Rohon-Beard neuron in a *trpa1b-/-* mutant with photo-activation performed on the cell body region of the cell. Red rectangular box indicates the time and location where photo-activation was performed. Larva was pretreated with 10 μM optovin.

Video S3. Time-lapse images of a zTrpa1b expressing Rohon-Beard neuron in a *trpa1b-/-* mutant with photo-activation performed on the neurite region of the cell. Red rectangular box indicates the time and location where photo-activation was performed. Larva was pretreated with 10 μM optovin.

Video S4. Optogenetic pacing of zebrafish hearts *in vivo* - optovin. Ventral views of a zebrafish heart at 2 dpf with different photo-activation frequencies. Photo-activation was performed at the atrium of the heart of a transgenic *trpa1b-/-* mutant larva expressing zTrpa1b in cardiomyocytes. The larva was pretreated with 10 μM optovin and microinjected with ivabradine into the pericardial space to inhibit pacemaker activity. The stimulation frequency and output heartbeat frequency are indicated on the individual panels (Simulation frequency / Heartbeat frequency).

Video S5. Optogenetic pacing of zebrafish hearts *in vivo* – 4g6. Ventral views of a zebrafish heart at 2 dpf with different photo-activation frequencies. Photo-activation was performed at the atrium of the heart of a transgenic *trpa1b-/-* mutant larva expressing zTrpa1b in cardiomyocytes. The larva was pretreated with10 μM 4g6 and microinjected with ivabradine into the pericardial space to inhibit pacemaker activity. The stimulation frequency and output heartbeat frequency were indicated on the individual panels (Simulation frequency / Heartbeat frequency).

Reference

1 Lam, P. Y., Fischer, R. S., Shin, W. D., Waterman, C. M. & Huttenlocher, A. Spinning disk confocal imaging of neutrophil migration in zebrafish. *Methods Mol Biol* **1124**, 219-233, doi:10.1007/978-1-62703-845-4_14 (2014).

Compound 11

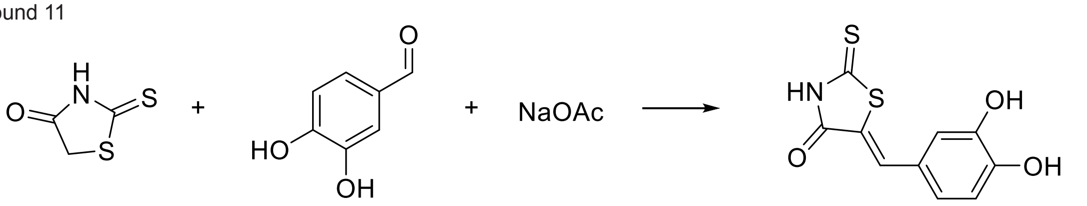

1H NMR (500 MHz, DMSO-d6) δ 13.63 (s, 1H), 9.92 (s, 1H), 9.50 (s, 1H), 7.45 (s, 1H), 6.98 (s, 1H), 6.97 (d, J = 8.0 Hz, 1H), 6.87 (d, J = 8.0 Hz, 1H). 13C NMR (126 MHz, DMSO-d6) δ 196.00 , 169.97 , 149.64 , 146.49 , 133.23 , 125.35 , 124.82 , 121.21 , 117.08 , 116.88 .

Compound 17

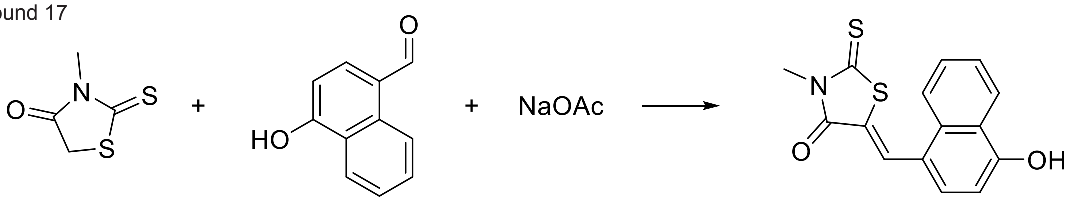

1H NMR (500 MHz, DMSO-d6) δ 11.26 (s, 1H), 8.41 (s, 1H), 8.25 (d, J = 8.3 Hz, 1H), 8.18 (d, J = 8.6 Hz, 1H), 7.72 – 7.66 (m, 1H), 7.63 – 7.55 (m, 2H), 7.07 (dd, J = 8.1, 1.4 Hz, 1H), 3.43 (s, 3H). 13C NMR (126 MHz, DMSO-d6) δ 194.37 , 167.27 , 157.65 , 133.56 , 130.33 , 129.92 , 128.74 , 126.17 , 125.11 , 123.42 , 123.28 , 121.25 , 120.68 , 109.10 , 31.60 .

Compound 19

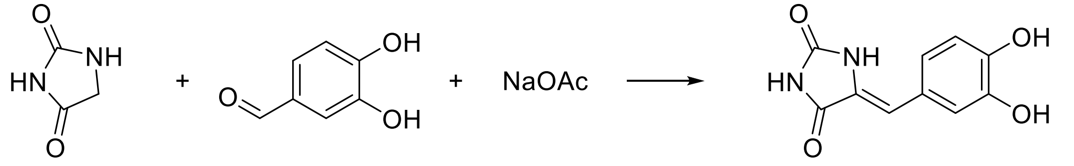

1H NMR (500 MHz, DMSO-d6) δ 11.07 (s, 1H), 10.24 (s, 1H), 9.42 (s, 1H), 8.95 (s, 1H), 6.96 (d, J = 7.9 Hz, 2H), 6.75 (d, J = 7.9 Hz, 1H), 6.25 (s, 1H). 13C NMR (126 MHz, DMSO-d6) δ 166.08 , 156.04 , 146.92 , 145.79 , 125.96 , 124.77 , 121.88 , 117.65 , 116.26 , 110.24 .

1 (Optovin)

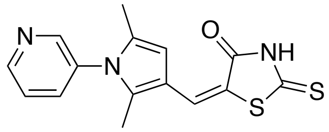

2 (4g6)

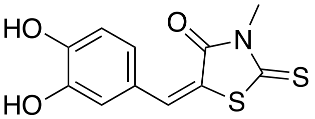

3

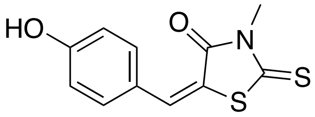

4

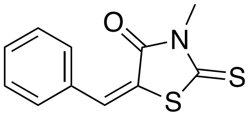

5

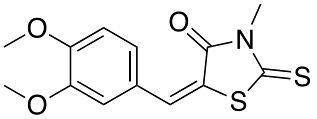

6

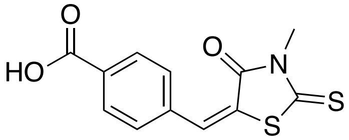

7

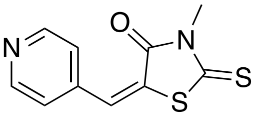

8

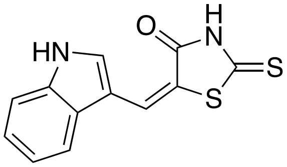

9

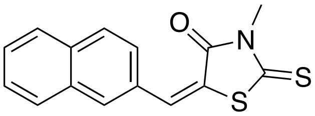

10

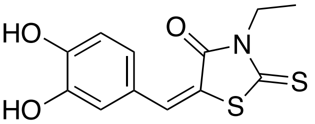

11

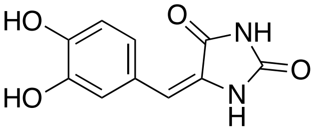

12

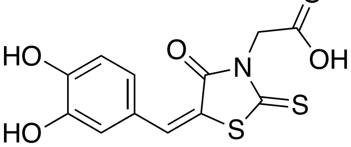

13

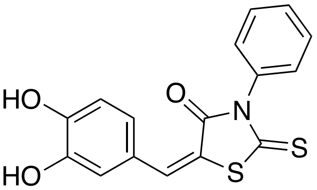

14

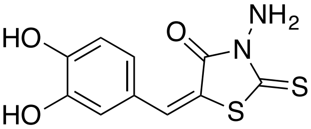

15

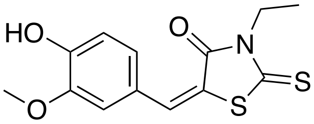

16

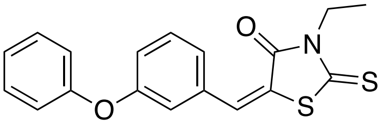

17

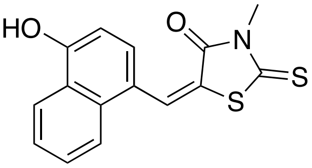

18

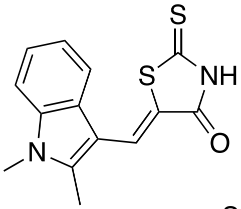

19

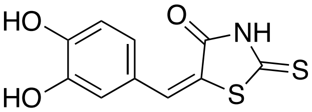

20

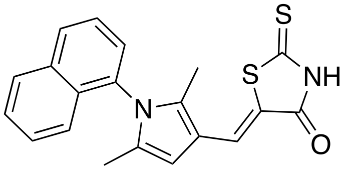

21

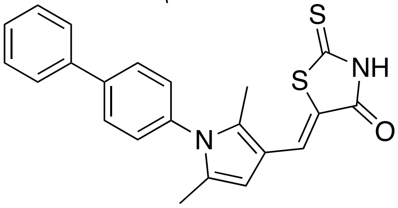

22

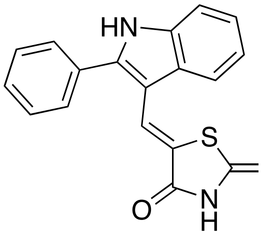

23

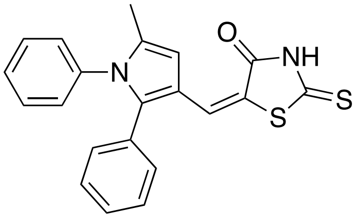

24

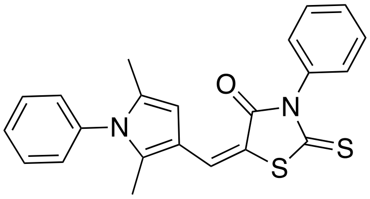

25

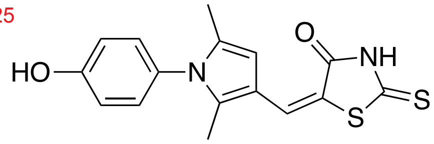

26

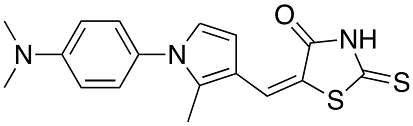

27

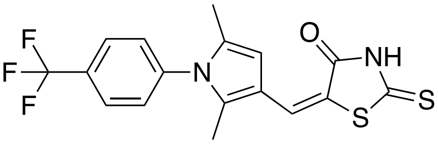

28

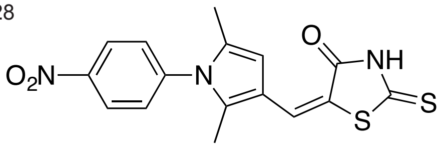

29

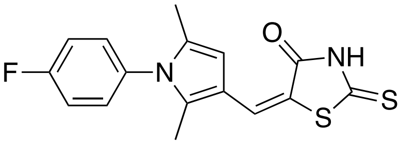

30

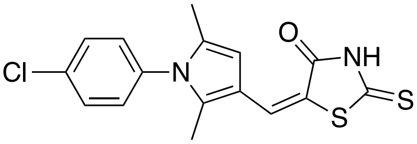

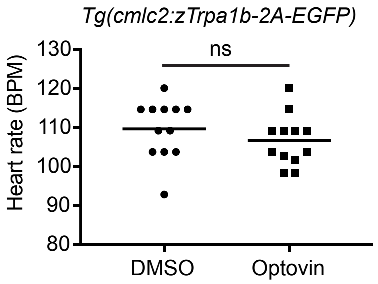

Tg(cmlc2:zTrpa1b-2A-EGFP)
ns
130
120
110
100
90
80
Heart rate (BPM)
DMSO
Optovin
